# Let there be Skyglow—light pollution from a large outdoor music festival (Lollapalooza Berlin 2016)

**DOI:** 10.1038/s41598-024-62448-7

**Published:** 2024-05-22

**Authors:** Andreas Jechow

**Affiliations:** 1 Department of Community and Ecosystem Ecology, Leibniz Institute of Freshwater Ecology and Inland Fisheries (IGB), Müggelseedamm 310, 12587 Berlin, Germany; 2 Department of Engineering, Brandenburg University of Applied Sciences, Magdeburger Str. 50, 14470 Brandenburg an der Havel, Germany

**Keywords:** Environmental impact, Urban ecology, Imaging techniques

## Abstract

Live music is often linked to elaborate light shows, particularly at large outdoor music festivals. However, artificial light at night is one form of environmental pollution, light pollution, and because outdoor festivals emit a substantial amount of artificial light into the environment, they are a potential source of light pollution. So far, no studies that quantified the impact of such festivals on urban light pollution and skyglow exist. Here, the light pollution produced by a major rock festival (Lollapalooza Berlin 2016 with 70,000 visitors per day in an urban park) was investigated with ground-based radiometry and night-time light data. A small night-sky radiometer installed near the main stages and a calibrated digital camera from a nearby observation spot inside of the park were used to quantify changes in night sky brightness and direct light emissions within the park. The impact of the music festival on the urban skyglow was indeed measurable. Zenith luminance increased locally by up to a factor of 8 and illuminance increased by about 50% at the observation spot within the park. The radiance detected by night-time satellite was also increased during the festival. This is the first time, that light pollution from such a major rock music event was quantified.

## Introduction

Live music shows are strongly linked to usage of artificial light. For example, the legendary Australian rock music band AC/DC altered the well-known biblical phrase “let there be light”^1^ in their iconic song “Let there be rock”^2,3^ to tell the story how rock music was created. In the second pre-chorus, they listed light as first item, before sound, drums and guitars. Nowadays, large outdoor music festivals have become very popular^4^ and contribute to the emission of artificial light at night (ALAN). ALAN is encroaching into nocturnal habitats, and it is growing in area and intensity^5,6^. Thus, it is more and more recognized as a form of environmental pollution, light pollution, which can affect a wide range of animals^7^ and plants^8^ as well as have an impact on biodiversity^9^. Artificial skyglow is one form of light pollution resulting from ALAN that is scattered back to the Earth’s surface within the atmosphere undergoing dynamics with changed atmospheric conditions^10–13^, snow cover^14^ or human usage^15,16^. Nowadays, either small night sky radiometers like the sky quality meter (SQM, see methods)^11,17^ or commercial digital cameras with fisheye lenses^17–21^ are popular for measuring the radiance of the night sky at zenith, often termed night sky brightness, and to extract light pollution including skyglow. As outdoor music festivals are also a source of ALAN, they are subsequently a potential source of light pollution including skyglow. However, no quantitative study that investigates light pollution of a music festivals exists so far.

In this work, the impact of a major rock music festival on urban skyglow is investigated at the Lollapalooza festival 2016 in Berlin. The festival was held in September 2016 in Treptower Park, a large urban park near the center of Berlin, and had 70,000 daily visitors for 2 days, including evenings. Permanent measurements of the evolution of the night sky radiance were obtained with an SQM mounted directly in front of one of the main stages of the festival, while wide-angle luminance measurements were performed with a DSLR camera and a fisheye lens from a nearby observation site. The results are set in the context of the urban skyglow by taking supporting measurements without the festival for clear and cloudy conditions. An increase of zenith radiance by more than a factor of 8 locally as well as an increase of more than 50% in both horizontal and vertical illuminance were found during the light check of the festival. This is the first time that light pollution and skyglow were investigated quantitatively in the context of a major music festival.

## Results

### Night sky radiance monitored at the festival site with a sky quality meter

**Figure 1:**
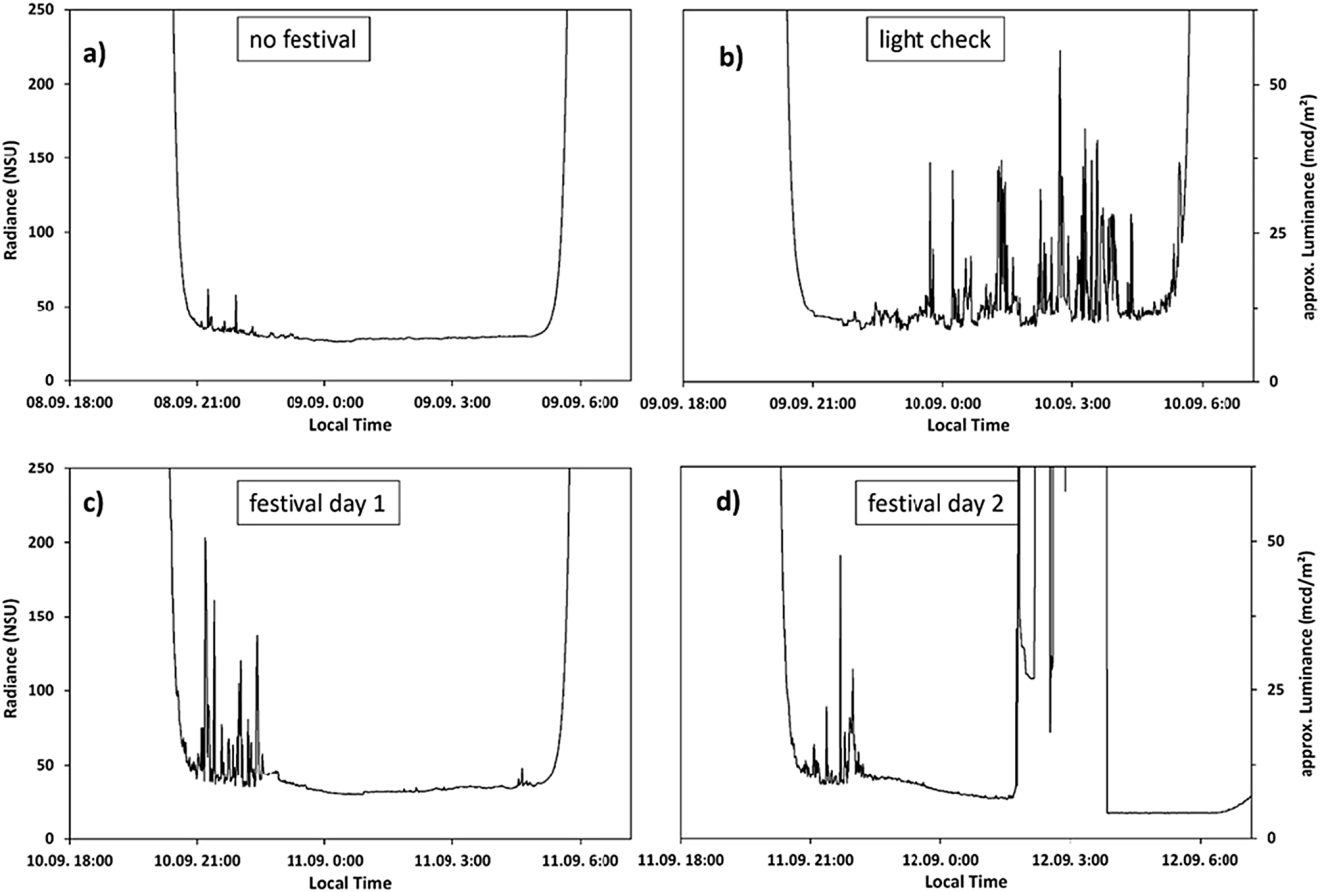
Radiance (left hand scale, in NSU, see methods) and approximated luminance (right hand scale, *see Methods for details) measured with the SQM at the FOH (front of house, see Methods) of main stage 1 of Lollapalooza festival 2016. Panel (**a**) shows the night sky radiance during a night before the festival where the ALAN emissions from the festival site were minimal, (**b**) shows the night sky radiance during a night before the festival when a light check was performed and (**c**) and (**d**) show the night sky radiance for the two festival nights, respectively.

Figure 1 shows the radiance in natural sky units (NSU, see methods) and approximated luminance (for details on NSU and luminance approximation see methods) measured with an SQM directly at the festival site, with the device mounted directly in front of one of the main stages (see Methods). The monitoring on site was done over four nights, which all had a clear sky, and started two nights before the festival on the 8th of September 2016.

**Figure 2:**
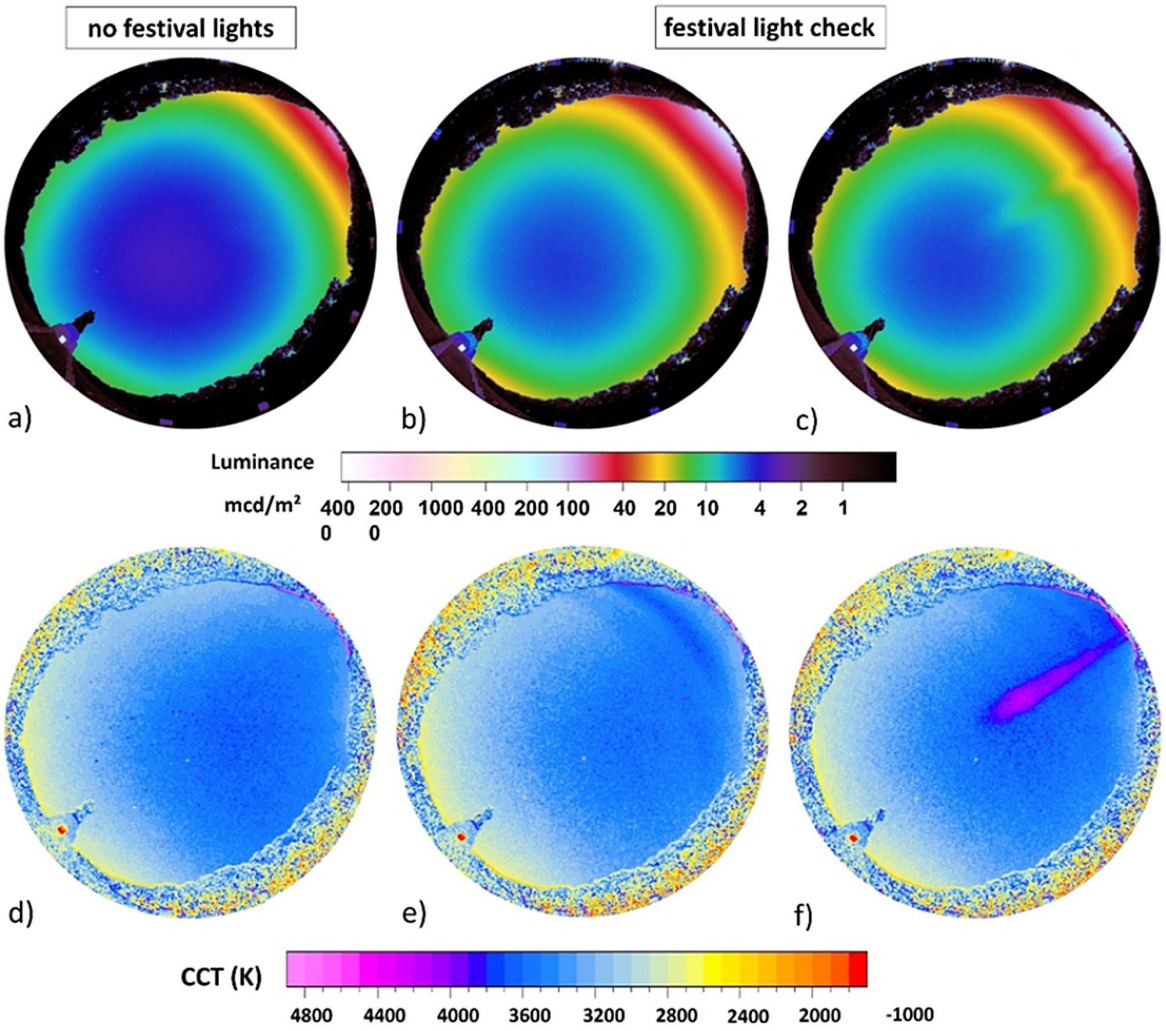
All-sky luminance (**a**–**c**) and CCT (**d**–**f**) maps obtained from the base of the soviet war memorial. Left-hand column shows data from a night without ALAN emission from the festival and the other two columns from the night with ALAN emission during the light check.

The radiance of first measurement night, two nights before the festival, is shown in Fig. 1a. This was the shortest of the four nights with astronomical night starting at 21:40 local time and ending at 4:28 the next morning. The moon was at 42.4% illumination and did set at 22:56 local time. At the start of astronomical night, it was 8° above the horizon. In this urban setting, the moon seemed to not have an obvious impact on the night sky radiance. The average radiance for this night (from 21:40 to 4:28) was 28.8 NSU (ca. 7.2 mcd/m^2^). The highest radiance measured during the astronomical night was 58.1 NSU (ca. 14.5 mcd/m^2^) obtained at 21:54 local time. The lowest radiance measured during astronomical night was 25.8 NSU (ca. 6.5 mcd/m). The highest radiance measured during twilight (21:15 local time) was 61.9 NSU (ca. 15.5 mcd/m).

The second night was the night just before the festival, when a light check of all artificial light sources of all stages was performed (Fig. 1b). The moon was at 52.3% illumination and did set at 23:35 local time. At the start of astronomical night, it was 12° above the horizon. The average radiance for this night (from 21:40 to 4:28) was 55.0 NSU (ca. 13.7 mcd/m^2^). The highest radiance measured during the astronomical night was 223 NSU (ca. 55.7 mcd/m^2^) obtained at 2:42 local time on 10th of September. For this night, the radiance during the twilight period in the evening remained elevated compared to the night before but had no peaks as the light check started to increase the brightness of the sky after 22:00. The lowest radiance measured during astronomical night was 34.4 NSU (ca. 8.4 mcd/m), which is well above the minimum radiance obtained the night before.

The third night (Fig. 1c) was the first festival night and bands played until 22:30. The moon was at 62.3% illumination and did set at 00:21 local time. At the start of astronomical night, it was 15° above the horizon. Like in the two nights before, the moon seemed to not have a strong impact on the night sky radiance measurements. However, there is a clear switch off of artificial lights at about 22:55 about 25 min after the last band was playing. The average radiance after the festival or for the later part of the night (from 22:55 to 4:28) was 33.1 NSU (ca. 8.3 mcd/m^2^) with the highest radiance being 39.8 NSU (ca. 10 mcd/m^2^) and the lowest 29.6 NSU (ca. 7.4 mcd/m^2^). During the festival and astronomical twilight and night (from 20:50 to 22:54), the average radiance was 55.2 NSU (ca. 13.8 mcd/m^2^) with the highest radiance being 203 NSU (ca. 50.8 mcd/m^2^) and the lowest 35.0 NSU (ca. 8.7 mcd/m^2^).

The last night (Fig. 1d) was the second festival night and bands played until 22:00. The moon was at 72.1% illumination and did set at 01:15 local time. At the start of astronomical night, it was 18° above the horizon. This time, the influence of moonlight on the radiance at zenith is perceivable, but by far not at the scale of the impact of artificial light originating from the festival. Again, the switch off after the bands stopped playing is visible and also the timepoint when the SQM was dismantled (1:40 in the morning, when radiance increased rapidly). After the festival (from 22:20 to 1:40) the average radiance was 33.2 NSU (ca. 8.5 mcd/m^2^) with the highest radiance being 43.3 NSU (ca. 10.3 mcd/m^2^) and the lowest 26.1 NSU (ca. 6.5 mcd/m^2^). During the festival and astronomical twilight and night (from 20:50 to 22:19), the average radiance was 49.0 NSU (ca. 12.2 mcd/m^2^) with the highest radiance being 190.5 NSU (ca. 47.6 mcd/m^2^) and the lowest 36.0 NSU (ca. 9.0 mcd/m^2^). The results from the SQM in relative radiance, SQM radiance and approximated luminance are summarized in Table 1.

**Table 1:** Radiance and approximated luminance for the different nights obtained with the SQM at the FOH tower.

Night	Rel. radiance (NSU)	Luminance* (mcd/m^2^)	SQM radiance (mag/arcsec^2^)
Night 1 (no festival)			
Avg	28.8	7.2	17.9
Max	58.1	14.5	17.2
Min	25.8	6.5	18.1
Night 2 (light check)			
Avg	55.0	13.7	17.2
Max	223	55.7	15.7
Min	34.4	8.4	17.8
Night 3 (after festival)			
Avg	33.1	8.3	17.8
Max	39.8	10.0	17.6
Min	29.6	7.4	17.9
Festival**			
Avg	55.2	13.8	17.2
Max	203	50.8	15.8
Min	35.0	8.7	17.7
Night 4 (after festival)			
Avg	33.2	8.5	17.8
Max	43.3	10.3	17.5
Min	26.1	6.5	18.1
Festival**			
Avg	49.0	12.2	17.4
Max	190.5	47.6	15.9
Min	36.0	9.0	17.7

*Luminance is only approximated, see methods for details. **Mainly during twilight.

### Radiance maps obtained with a fisheye camera

**Figure 3:**
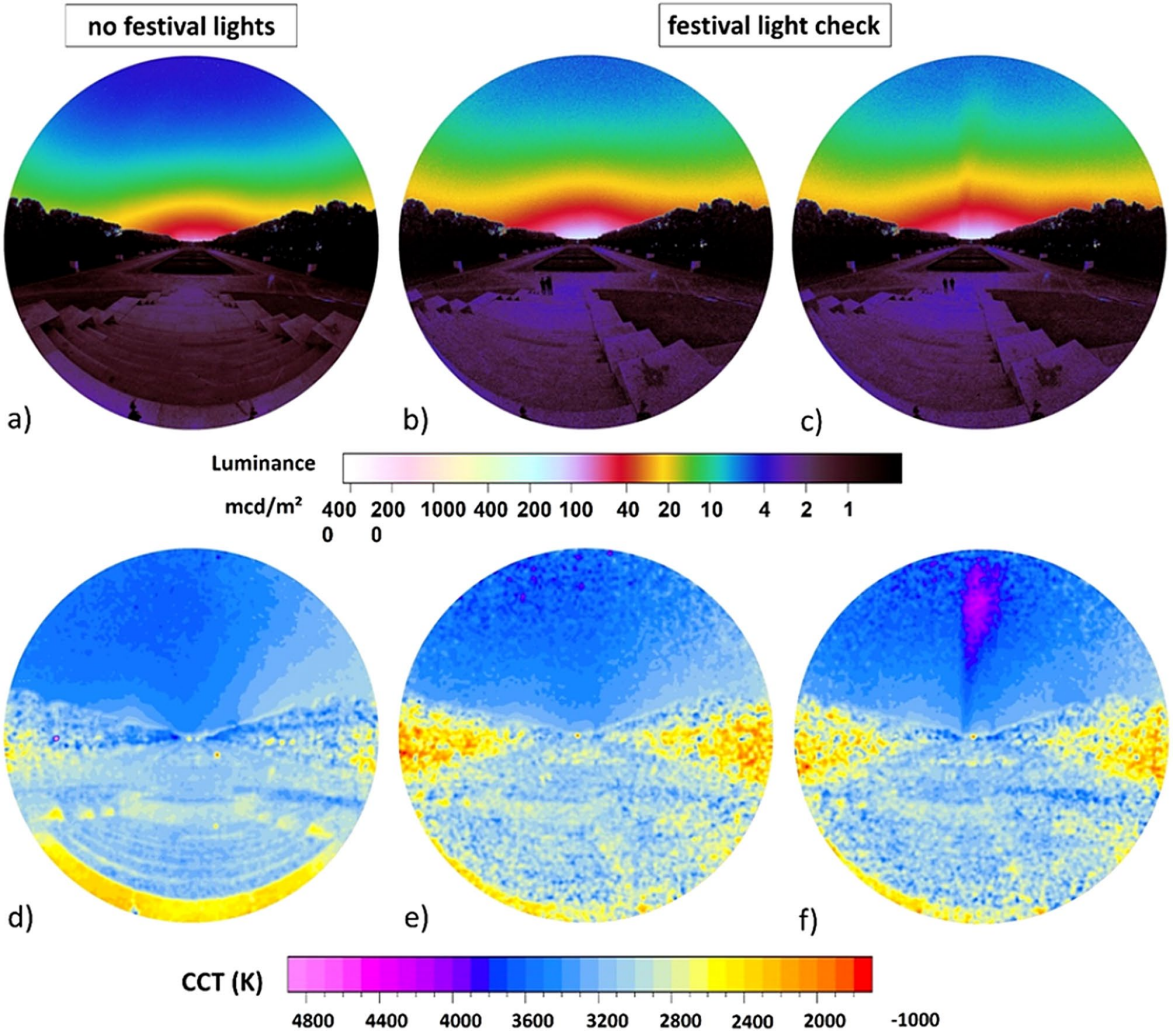
Vertical plane luminance (**a**–**c**) and CCT (**d**–**f**) maps obtained from the base of the soviet war memorial. Left-hand column shows data from a night without ALAN emission from the festival and the other two columns from the night with ALAN emission during the light check.

Figure 2 shows the calculated luminance (a–c) and CCT (correlated color temperature) maps (d–f) obtained from all-sky images taken from the base of the war memorial and Fig. 3 shows analogously luminance and CCT maps from vertical plane images obtained from the pedestal of the war memorial (see methods). In both datasets, the left-hand column shows the first measurement night before the festival without ALAN emission from the festival, and the middle and right-hand column show the second measurement night with substantial ALAN emission during the light check. The right-hand site in both datasets show the presence of a spotlight shining across the sky.

From the all-sky images (Fig. 2) the luminance at zenith (SQM feature of SQC), the (cosine corrected) horizontal illuminance and the hemispheric scalar illuminance were calculated^21^. Without ALAN from the festival (Fig. 2a), 01:26 am 09.09.2016, the luminance at zenith was 5.2 mcd/m^2^ (18.3 mag/arcsec^2^), horizontal illuminance was 19.5 mlx and scalar illuminance was 40.5 mlx. During the measurement with ALAN from the light check of the festival (23:20–23:45 on 09.09.2016), the luminance at zenith was 7.5 mcd/m^2^ (17.9 mag/arcsec^2^) on average (44% brighter than the night before). The horizontal illuminance was 29.4 mlx on average (51% brighter than the night before) and the scalar illuminance was 62.8 mlx on average (55% brighter than the night before). For the whole sky, CCT was not measurably differing in any of the scenarios but the spot-light with higher CCT is clearly visible in Fig. 2f.

From the vertical plane images (Fig. 3) the luminance near the horizon (10° circle, 8° altitude), the (cosine corrected) vertical illuminance and the hemispheric scalar illuminance were calculated. Without ALAN from the festival (01:35 am 09.09.2016), the luminance at the horizon was 39.1 mcd/m^2^, vertical illuminance was 22.0 mlx and scalar illuminance was 34.7 mlx. During the measurement with ALAN from the light check of the festival (23:20–23:45 on 09.09.2016), the luminance at zenith was 64.8 mcd/m^2^ on average (> 65% brighter than the night before), with a maximum of 72.4 mcd/m^2^ and a minimum of 61.1 mcd/m^2^. The horizontal illuminance was 34.2 mlx on average (> 55% brighter than the night before) with a maximum of 35.8 mlx and a minimum of 31.9 mlx. The scalar illuminance was 63.1 mlx on average (53% brighter than the night before) with a maximum of 64.8 mlx and a minimum of 60.3 mlx. Again, a difference in CCT was only detectable for the spotlight (Fig. 3f).

### Night-time lights

**Figure 4:**
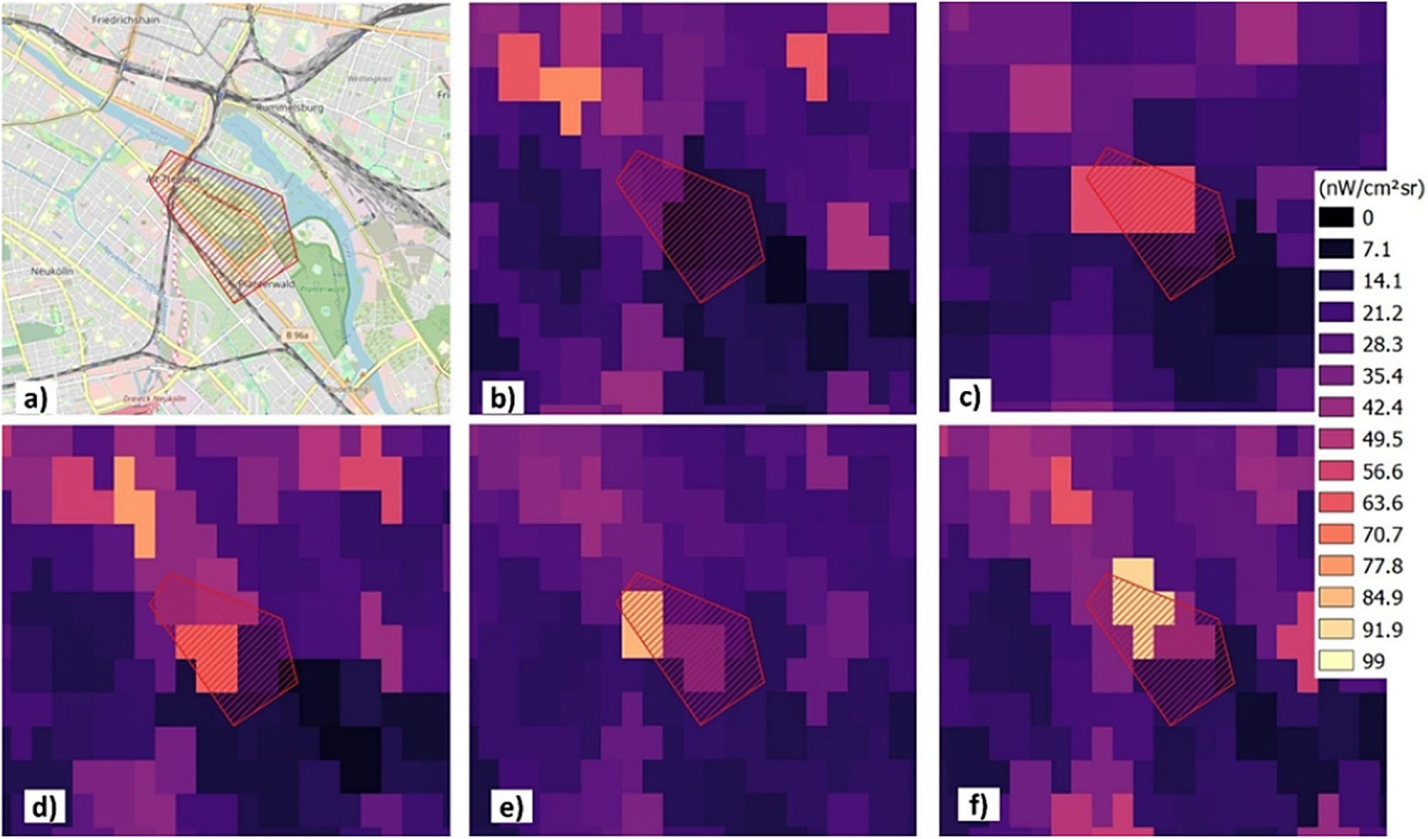
(**a**) Map of Treptower Park showing the festival polygon b)-f) VIIRS DNB radiance for (**b**) a night after the festival, (**c**) a night during construction, (**d**) the night of the light check, (**e**) festival night 1 and (**f**) festival night 2.

Figure 4 shows a map of Treptower Park (a) and the VIIRS DNB data (b–f) for several nights with a red box indicating the exact festival site and the polygon used for zone statistics. The situation without festival on 01.10.2016 is shown in (b), the night during construction (09.09.2016) is shown in (c), the night of the lightcheck (10.09.2016) is shown in (d) and the two festival nights (11 and 12.09.2016) are shown in (e) and (f), respectively. The results from the zone statistics analysis are summarized in Table 2 showing the pixel radiance means and the standard deviation as uncertainties. During construction and the festival, a clear increase in radiance is observed from the satellite. For comparison, an area near Berlin without ALAN emission, the Schorfheide (52° 56′ 45.6″ N 13° 30′ 19.5″ E) was analyzed that showed no such change.

**Table 2:** VIIRS DNB radiance (in nW/cm^2^sr) for the different nights shown in Fig. 4 for the festival site and a dark reference outside of Berlin.

Location	No festival	Construction	Lightcheck	Festival 1	Festival 2
	(01.10.2022)	(09.09.2022)	(10.09.2022)	(11.09.2022)	(12.09.2022)
Lollapalooza	15.2 ± 7.2	38.2 ± 24.0	37.8 ± 22.2	35.7 ± 23.7	41.4 ± 29.1
Schorfheide	0.1 ± 0.1	0.2 ± 0.3	0.1 ± 0.1	0.1 ± 0.4	− 0.1 ± 0.3

## Discussion

This is the first time that the light pollution produced by a large music festival was quantified with ground based-measurements and satellite data. The ground-based measurements show that the emissions of ALAN from the festival brighten the sky at and near the festival site, which leads to a significant increase of illuminance in the surroundings. Directly at the FOH tower, next to the main stages, an enhanced night sky radiance of more than 8 times higher than without festival was determined during the light check and the festival itself. While the radiance measurements during the festival could have been falsified by a dust cloud produced by a large crowd at dry weather conditions, this can be ruled out for the light check performed the night before the festival. Such a dust cloud would have added an additional layer of light scattering particles. However, during the light check no crowd was present at the festival site and no peculiar weather like strong winds did produce any dust cloud. There was a slight deviation between the camera measurements obtained at a distance of more than 600 m from the festival site at the soviet war memorial and the SQM measurements directly at the site. This could mean, that some light that is propagating horizontally does not reach the neighboring site (there are some trees blocking the direct light from the festival site). Also, the spectral mismatch between the DSLR camera and the SQM could induce such deviation. Nevertheless, luminance at zenith, near the horizon, as well as both, horizontal and vertical illuminances obtained at a distance of more than 600 m from the festival site at the soviet war memorial were elevated by 50% or more during the light check compared to the night without light check. This is surprising, because the urban skyglow without festival has already elevated the horizontal illuminance at Treptower Park by about a factor of 20 compared to a pristine rural location without any ALAN or skyglow^10^. For comparison, the illuminance at such a rural location would be on the order of 1 mlx. This leads to the assumption, that the relative impact of ALAN from a similar festival is larger when the festival site is in a suburban, peri-urban or even in a pristine rural location as the light emissions will be on the same order, but the background brightness is much lower.

The satellite data show an increase in radiance at the festival site for the time of the festival and during construction. Radiance increased to up to 41.4 ± 29.1 nW/cm^2^sr during the festival compared to 15.2 ± 7.2 nW/cm^2^sr without the festival. During the same period, the dark reference site remained basically constant at near zero values. Thus, the festival was detectable despite the non-ideal overpass times and the intrinsically angular deficiency of the satellite method, which is unable to detect ALAN propagating at angles far from nadir or zenith, respectively^22^.

It is worth noting that for this specific festival the ALAN emission during the festival itself was strongly linked to the curfew at 22:30 and 22:00, respectively. This most likely also affected the satellite data, which were overpassing at 2:30 am. This fact, and the aforementioned problem of lack of angular resolution of the satellite, makes it difficult to utilize just satellite data for such studies. Ground-based measurements can detect light that is scattered from light emitted at angles far from zenith and SQMs have the advantage of a high temporal resolution. Therefore, the SQM data shows a higher variation than the satellite data, particularly during the night of the light check. Unfortunately, no data from the SQM is available without any festival related lights (outside of the construction time).

The curfew was most likely a condition against noise pollution for the local residents. Thus, during the two festival nights ALAN was strongest during the twilight hours and early night as well and ALAN emission dropped back to before festival condition later at night. However, the excessive light check just during the night before the festival suggests that no such condition existed towards a restriction of light emissions. It was awkward to be at the empty festival ground without any sound but with a full light show. Furthermore, there was substantial usage of ALAN already long before the music event itself. During the construction of the festival site lights were used at night which happened also during the night and when the crew finished their work, the construction lights remained switched on due to security concerns.

In conclusion, a major music festival is a source of ALAN and therefore light pollution. Skyglow and illuminance were measurably elevated even in an urban context. It seems, that neither the construction phase nor the festival itself had a restriction on light emission at a certain night-time, which might be simply neglected in policy making in favor to the obvious human centric noise pollution regulations. Future research could cover the study of festivals in a non-urban context, include aerial imaging data, ideally also at different observation angles^22^ or from drones^23^ and also ground based-measurements at different distances^10^ ideally with multiple instruments in parallel^24^.

## Materials and methods

### The festival

**Figure 5:**
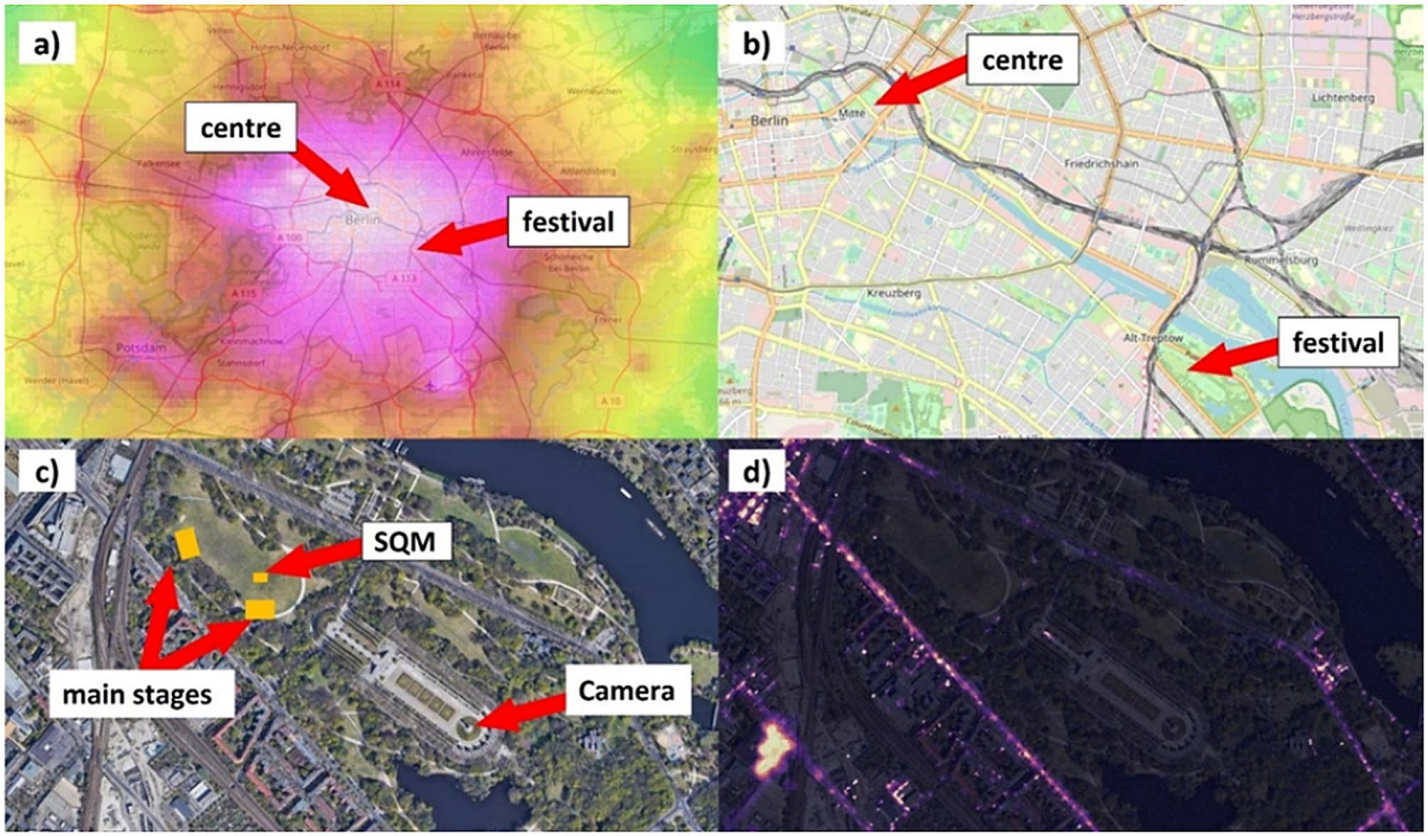
Map of the festival site and measurement device locations. (**a**) Skyglow data from Falchi et al.^5^ for the Berlin metropolitan area, (**b**) map of the area indicating the festival location with respect to Berlin city center, (**c**) map of Treptower Park and the measurement sites (**d**) same map as (**c**) but overlaid with aerial night-time light data from Kuechly et al.^26^. Figure produced with QGIS 3.10 using Open Street Map (**a**, **b**) and google (**c**, **d**).

The Lollapalooza 2016 festival in Berlin, Germany, was a major two day indie, pop, rock, hip hop and electronic festival, held on 10th of September and 11th of September 2016. The festival was sold out with 70,000 visitors per day and featured international top acts like Kings of Leon and Radiohead. The festival (see Fig. 5 for site description) was held in Treptower Park only in that year because there was substantial resistance by local residents, nature conservation groups and other stakeholders because the park is a garden monument, a war memorial and burial site and hosts a significant proportion of urban wildlife and particularly nocturnal hedgehogs caught public attention^25^. There were strict curfews at 22:30 local time on Saturday the 10th, and 22:00 on Sunday the 11th.

### Measurement sites

**Figure 6:**
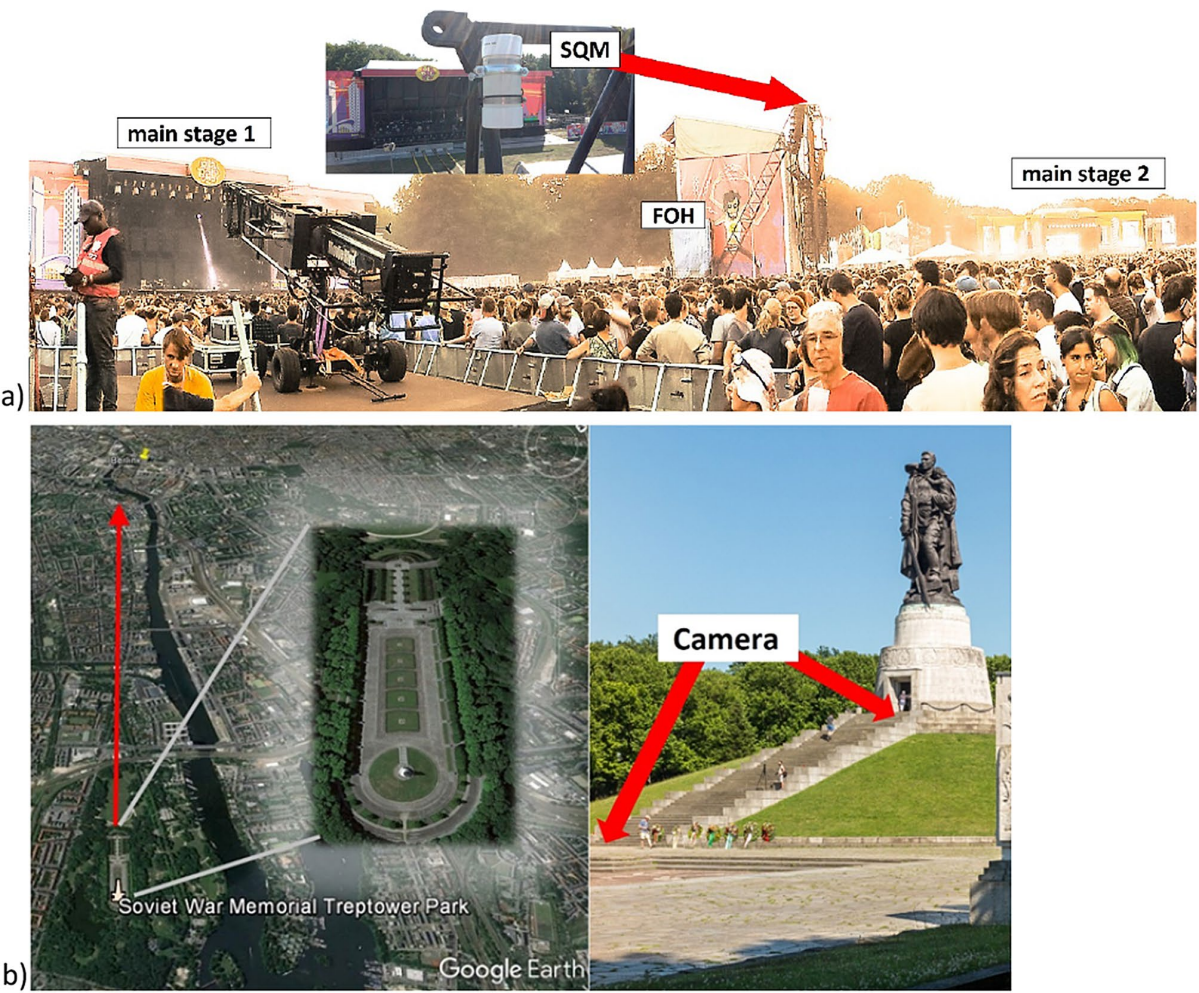
(**a**) Position of the Sky Quality Meter (SQM) at the festival site. The device was mounted at the front of house (FOH) tower in front of main stage 1 of the festival. (Image by Andreas Jechow). (**b**) Camera position and viewing direction towards the city centre and festival site at the Soviet War Memorial within Treptower Park. (Lefthand images from GoogleEarth, righthand image by JoachimKohler-HB from wikicommons licensed under CC BY-SA 4.0).

Measurements were conducted at the main festival site, see Figs. 5c and 6a, and at the Soviet War Memorial in Treptower Park, an urban park in Berlin. The Treptower Park (52° 29′ 18.6″ N 13° 28′ 11.1″ E) lies about 6 km southeast of the city center of Berlin. The park was laid out in 1888 and has an area of 0.88 km^2^. The Soviet War Memorial within the park (52° 29′ 10.1″ N 13° 28′ 19.1″ E) is a 12 m high statue built 1949 on an 18 m high pedestal (Fig. 2 upper right). The viewing axis from this pedestal (Fig. 6b) is facing almost directly towards the city center. The memorial is fenced and lies somewhat secluded within the park. It is not frequently visited by tourists during the night. The area of the memorial is almost unlit apart from the statues bottom that hosts a slightly lit pavilion. The site is lined by trees that shield most light from surrounding streets at least during the summer. During the festival, this site was not accessible.

## Measurement instruments

### Sky quality meter

The sky quality meter (SQM; Unihedron, Grimsby, Ontario, Canada), a small radiometer with a spectral response that is close to but not exactly photopic nor the astronomical Johnson V band is designed to measure the radiance of the night sky at zenith, often termed night sky brightness. The SQM version with an integrated lens (SQM-L) measures sky radiance (or near luminance) for a patch of the sky with an opening angle of 20°^17^. The SQM provides the night sky radiance in units of (SQM) magnitudes per square arc second (mag(SQM)/arcsec^2^) which is a logarithmic scale that decreases with increasing brightness. A decrease of 5 mag(SQM)/arcsec^2^ equals an increase in radiance (or near luminance) by a factor of 100. Often, a value of 21.6 mag(SQM)/arcsec^2^ is used as a reference value for a typical moonless clear night with only natural contributions to the night sky radiance^18^, sometimes a value of 22.0 mag(SQM)/arcsec^2^ is given as a minimum^5^. Due to the spectral mismatch (see^17^ for a review comparing devices), the sky radiance ${L}_{SQM,zen}$ measured with an SQM can only approximately be converted to a sky luminance at zenith ${L}_{v,zen}$ using ${L}_{v,zen}\approx 10.8 \cdot {10}^{4} \cdot {10}^{-0.4 \cdot {L}_{SQM}}$. To provide a more intuitive comparison between different night sky radiances for non-specialist, ${L}_{SQM,zen}$ are sometimes transformed to the so-called natural sky units (NSU)^17^. One NSU indicates how much brighter or darker a night sky radiance is compared to the reference value 21.6 mag(SQM)/arcsec^2^ (which is approximately a luminance of about 0.25 mcd/m^2^). It is worth pointing out that the SQM and the unit conversions are not perfect at all, but currently this is the most commonly used instrument for night sky radiance monitoring and the NSU definition used here is also the most widespread.

A sky quality meter with a lens and data logger (SQM-LU-DL) was used to track the changes in night sky radiance at the festival site itself over the course of four nights. The SQM was mounted on top of the front of house (FOH) tower of main stage 1 as shown in Fig. 6a). It was not straightforward to mount the device at this height at a festival site with many visitors and it required a thorough security screening by the organizers which was allowed last minute, and this set the relatively short measurement interval. The SQM was located in the standard weatherproof housing available from the manufacturer. The measurement interval was set to 1 min.

### DSLR camera and image processing software

Calibrated digital cameras equipped with a fisheye lens have been proven to be valuable for assessment of dark sky parks^18–20,27^ urban sites ^16^ or ecological sites even for challenging outdoor fieldwork^14,21^. Here, hemispherical images were obtained from (or near) the Soviet War Memorial in Treptower Park as shown in Fig. 2b. All-sky images were obtained by positioning the camera in front of the statue and pointing the camera towards the zenith. Vertical plane images were obtained by positioning the camera on top of the pedestal of the statue and aligned along the viewing axis of the Soviet War Memorial, pointing the camera horizontally towards the city center. Imaging data was acquired on several nights before the festival. Access during the festival was not allowed due to local government constraints. Images were obtained with a commercial DSLR camera, a Canon EOS 6D, which has a full-frame 20.2 Megapixel (5496 × 3670) CMOS sensor. The camera was mounted on a tripod and operated with a circular fisheye lens (Sigma EX DG with 8 mm focal length) always at full aperture of 3.5. For image processing the commercial “sky quality camera” software (Version 1.8, Euromix, Ljubljana, Slovenia) was used. The camera was calibrated by the software manufacturer, including radiometric (and like the SQM near photometric calibration) using the green channel of the camera, as well as correction of optical aberrations like vignetting. The software calculates the sky radiance (and luminance again with a spectral mismatch) ${L}_{v,sky}$ for each camera pixel. Furthermore, the software allows to acquire the cosine corrected illuminance in the imaging plane and scalar illuminance of the hemisphere (see Ref^21^ for details on calibration, mismatch and illuminance calculations). From the three spectral channels, CCT is calculated by the software. The software provides luminance and CCT maps, allows the subtraction of one image from another, to analyze sectors of the image, and to do cross sections.

### Night-time light data

Nightly mosaics of night-time light data from the Suomi National Polar-orbiting Partnership (NPP) instrument Visible Infrared Imaging Radiometer Suite (VIIRS) Day-Night Band (DNB) were used for the analysis which is freely available at the website of the Earth Observation Group at Payne Institute^28^. During summer, the VIIRS overpass is at 2:30 local time. The measurement nights (09.09.2016–12.09.2016) plus a moonless night after the festival (01.10.2016) were downloaded as GeoTIFFs in rade9 (in units W/cm^2^ sr) and processed in QGIS (Version 3.10)^29^ using the plugin zone statistics. Polygons were created with the shape of the festival area and an area north of Berlin without ALAN emission, the Schorfheide (52° 56′ 45.6″ N 13° 30′ 19.5″ E), as reference to check for potential offset in the VIIRS DNB nightly data.

## Data Availability

All data are available on reasonable request from the corresponding author (andreas.jechow@th-brandenburg.de).
